# Molecular basis for functional switching of GFP by two disparate non-native post-translational modifications of a phenyl azide reaction handle†
†Electronic supplementary information (ESI) available. See DOI: 10.1039/c6sc00944a



**DOI:** 10.1039/c6sc00944a

**Published:** 2016-06-29

**Authors:** Andrew M. Hartley, Harley L. Worthy, Samuel C. Reddington, Pierre J. Rizkallah, D. Dafydd Jones

**Affiliations:** a School of Biosciences , Cardiff University , Cardiff , UK . Email: jonesdd@cardiff.ac.uk; b School of Medicine , Cardiff University , Cardiff , UK

## Abstract

Through the genetic incorporation of a single phenyl azide group into superfolder GFP (sfGFP) at residue 148 we provide a molecular description of how this highly versatile chemical handle can be used to positively switch protein function *in vitro* and *in vivo via* either photochemistry or bioconjugation. Replacement of H148 with *p*-azido-l-phenylalanine (azF) blue shifts the major excitation peak ∼90 nm by disrupting the H-bond and proton transfer network that defines the chromophore charged state. Bioorthogonal click modification with a simple dibenzylcyclooctyne or UV irradiation shifts the neutral-anionic chromophore equilibrium, switching fluorescence to the optimal ∼490 nm excitation. Click modification also improved quantum yield over both the unmodified and original protein. Crystal structures of both the click modified and photochemically converted forms show that functional switching is due to local conformational changes that optimise the interaction networks surrounding the chromophore. Crystal structure and mass spectrometry studies of the irradiated protein suggest that the phenyl azide converts to a dehydroazepine and/or an azepinone. Thus, protein embedded phenyl azides can be used beyond simple photocrosslinkers and passive conjugation handles, and mimic many natural post-translational modifications: modulation though changes in interaction networks.

## Abstract 2

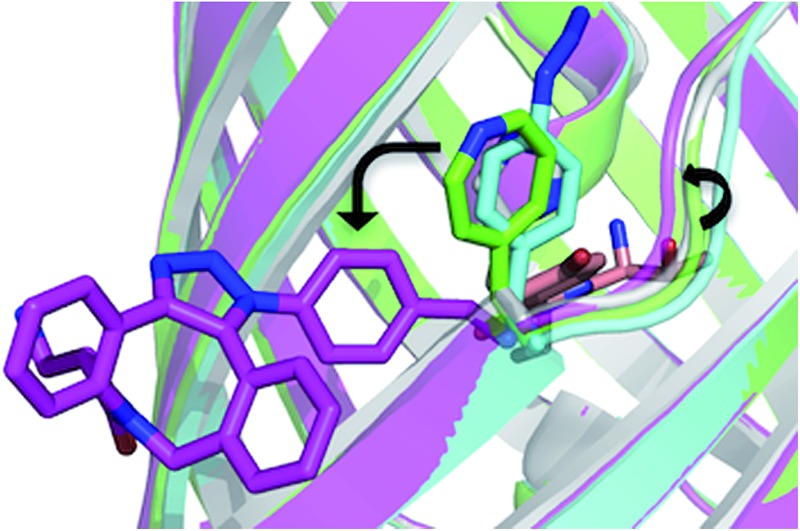
Through the genetic incorporation of a single phenyl azide group into superfolder GFP (sfGFP) at residue 148 we provide a molecular description of how this highly versatile chemical handle can be used to positively switch protein function *in vitro* and *in vivo via* either photochemistry or bioconjugation.

## Introduction

**Fig. 1:**
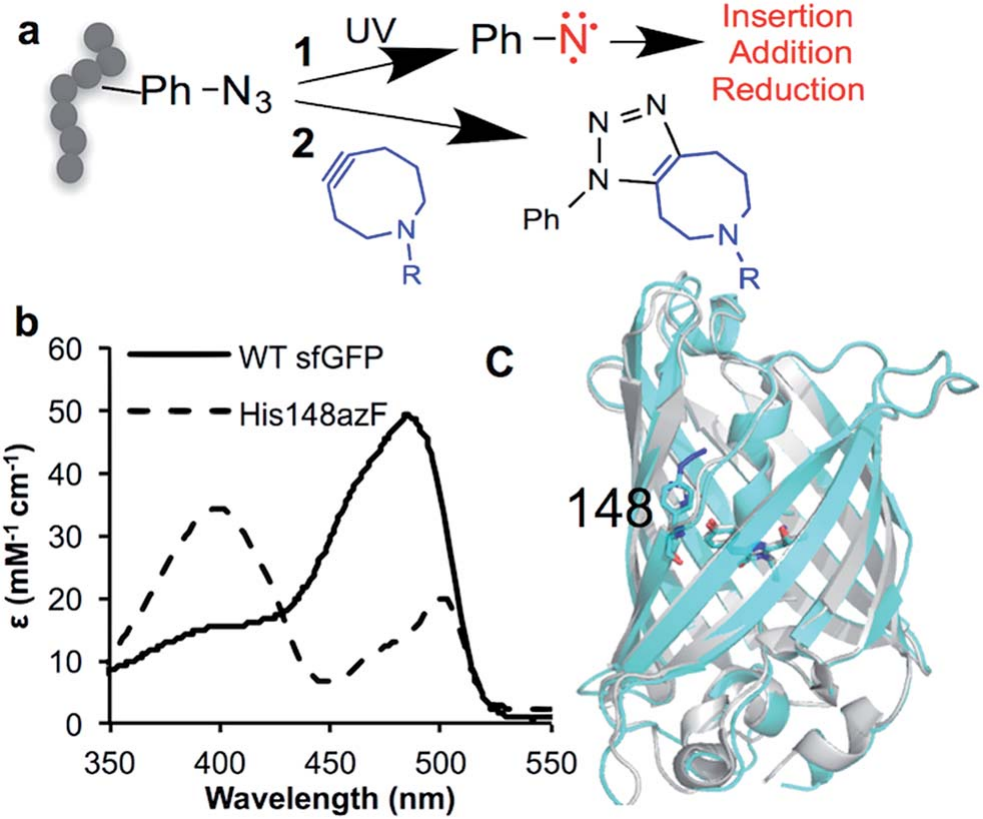
Incorporation of phenyl azide chemistry into sfGFP. (a) Routes for modulating protein activity *via* nnPTM; 1 – photochemistry, 2 – SPAAC addition (b) switch in sfGFP (black line) absorbance on mutating H148 to azF (dashed line). (c) Crystal structure of dark state sfGFP^148azF^ (cyan) overlaid with sfGFP (PDB ; 2B3P^26^).

Expansion of the genetic code (see^1–4^ for recent reviews) has opened up new approaches for precise post-translational modification (PTM) through the targeted incorporation of non-canonical amino acids (ncAA) with useful reactive handles.^5,6^ In natural biological systems, PTM is pivotal for modulating and supplementing protein function;^7^ among the most important are permanent covalent modifications with their impact ranging from the introduction of new non-proteinaceous chemical features to induced conformational change. Protein modification using genetically encoded non-native chemistry has been used primarily for passive labelling with suitable reporter adducts (see^5,8,9^ for recent reviews) rather than to modulate function. One versatile ncAA is azidophenylalanine (azF), as it allows incorporation of phenyl azide chemistry (a classic tool in biochemistry^10–12^) into proteins.^13,14^ Phenyl azide has two distinct chemistries that makes it particularly useful in terms of new PTM approaches: photochemical activation and bioorthogonal click chemistry (Fig. 1a). On irradiation with UV light, the azide loses N_2_ to form a reactive singlet nitrene that can sample various pathways (Fig. 1a; see ESI Scheme S1† for detailed description),^15^ resulting in changes to protein function.^16–19^ Alternatively, it can be used in strain promoted azide–alkyne cycloaddition (SPAAC^20,21^) (Fig. 1a). SPAAC is a biocompatible and bioorthogonal reaction that is quickly emerging as a powerful approach to label biomolecules, including proteins, with various adducts both *in vitro* and *in vivo*.^1,6,22,23^ Genetic encoding of azF^13^ allows highly precise placement of phenyl azide chemistry opening up the opportunity to actively modulate of protein function *via* these two novel approaches. While a few studies have emerged recently^17,18^ concerning the molecular basis of photochemical control, little is known beyond theoretical modelling^24^ how attachment of click reagents such as cyclooctynes influence protein structure; both aspects thus lack a detailed molecular basis of action that could potentially aid future application with regards to new and general approaches to protein functional modulation.

We have used superfolder GFP (sfGFP) as a model system to exemplify and understand mechanism of action of these two different nnPTM routes.^18,25^ SfGFP^26^ is an intrinsically fluorescent protein derived from the original *Aequorea victoria* GFP.^27^ Autofluorescent proteins such as GFP have proved to be an excellent model system for investigating and understanding the effect of ncAA incorporation on protein function (for examples see reviews^2,28^); structural studies are crucial for understanding the molecular basis of action of ncAA engineering but are still relatively rare,^10,16,29–33^ and even less so for ones that actively modulate function. Here we provide a timely structural insight and comparison concerning fluorescence switching on either click addition or irradiation of sfGFP containing the H148azF mutation (sfGFP^148azF^). The structure of the DBCO modified sfGFP^148azF^ (the first such structure of a SPAAC modified protein to our knowledge) reveals an induced conformation change that drives the functional switch potentially opening up SPAAC bioorthogonal conjugation as an approach for modulating proteins rather than simply labelling them. On irradiation rather than acting as a classical photocrosslinker, functional switching is achieved through local conformational rearrangements on end-product formation.

## Results and discussion

### Structural effect of H148azF mutation

**Fig. 2:**
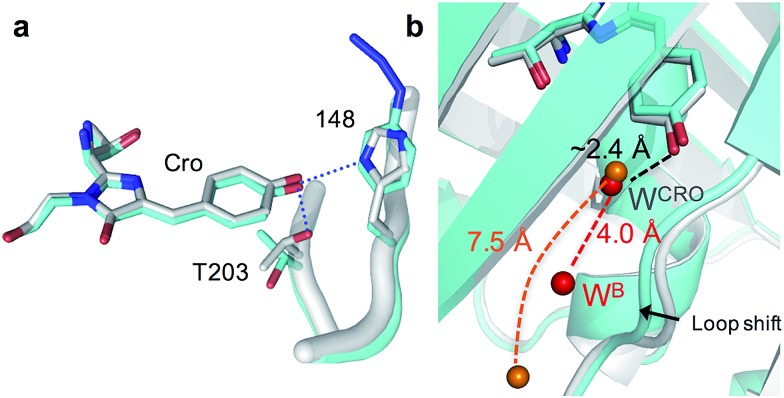
Structural changes on mutating H148 to azF. Original sfGFP and sfGFP^148azF^ are coloured grey and cyan respectively. (a) The chromophore (Cro) with T203 and 148 highlighted (H-bonds in sfGFP are shown as blue dots and absent in sfGFP^148azF^). The terminal N_2_ group of azF148 represents a modelled structural representation. (b) Positions of water molecules (W) in sfGFP^148azF^ (orange balls) and original sfGFP (red balls).

H148 plays an important role in defining the fluorescence properties of GFP through the contribution of the imidazole side chain to the charge transfer network that modulates chromophore protonation state equilibrium,^34–38^ and maintaining local structural integrity.^39,40^ Thus, it represents an excellent target to assess how ncAA can be used to influence critical interactions that define function. Replacement of H148 with azF results in a ∼90 nm blue shift in the major absorbance peak to the less favourable 400 nm (Fig. 1b and Table 1), indicative of the chromophore (Cro) ground state equilibrium being dominated by protonated (neutral) form.^36^ The sole emission peak at ∼511 nm is retained (Fig. S1†) confirming excited state proton transfer. The crystal structure of sfGFP^148azF^ (Fig. 1c and Table S1;† PDB code ; 5BT0) indicates that propagated changes in the local interactions on mutation are responsible for the change in fluorescence.¶
¶The crystal structure have been deposited in the PDB with the following codes: 5BT0, 5DY6, 5BTT. No electron density was observed past the first nitrogen position of the azide group despite mass spectrometry confirming its presence prior to crystallisation (Fig. S2† and Table 2). The likely explanation for the absence of N_2_ is that the azide moiety accesses multiple conformations through rotation but we cannot rule out loss of N_2_ during X-ray bombardment during data collection. We have modelled in an accessible configuration based on previous work^24^ that would maintain the predicted bond angles of azF with minimal steric hindrance (Fig. 2).

**Table 1:** Spectral properties of sfGFP^148azF^

	*λ*_max_ (nm)	*λ*_em_ (nm)	*ε* (M^–1^ cm^–1^)	QY^*d*^ (%)	Brightness	
					M^–1^ cm^–1^	% sfGFP
Dark^*a*^	400	511	34 246	69	23 630	64
	500	511	15 769	32	5046	14
UV	400	511	24 024	30	7207	20
	490	511	34 262	51	17 474	48
+DBCO-amine	400	511^*b*^	23 600	N/D	N/A	
	490	511	36 900	85	31 365	85
sfGFP^*a*^	485	511	49 036^*c*^	75	36 777	100

^*a*^Values calculated previously by Reddington *et al.* ^18^

^*b*^Observed emission was negligible (Fig. 3b).

^*c*^ *ε* calculated for sfGFP in our lab,^18^ which differs from the previously reported value of 83 300 M^–1^ cm^–1^.^26^

^*d*^Quantum yield.

**Table 2:** Calculated and measured mass for the different sfGFP^148azF^ modification events

Species	Calculated^*a*^ *M*_w_ (Da)	Measured^*b*^ *M*_w_ (Da)
sfGFP^148azF^	27 879	27 877
sfGFP^148azF^ DBCO-amine [+276 Da]	28 155	28 154

**Photolysis product (no crosslink)**		
Phenyl amine^*c*^	27 853	27 865
Dehydroazepine^*c*^	27 851	
Azepinone^*c*^	27 867	

^*a*^Mass calculated using the ExPasy *M*_w_ tool (http://web.expasy.org/compute_pi/) for the native protein sequence corresponding to sfGFP^H148Y^ and then adjusted for chromophore maturation (–18 Da) and replacement of Y with azF (+25).

^*b*^See Fig. S2, S5 and S9 for mass spectra.

^*c*^See Scheme S1 for chemical structures.

The H148azF mutation removes two H-bonds from the Cro hydroxyl group to: (1) the original 148 imidazole and; (2) the OH side chain of T203 (Fig. 2a). Both these H-bonds are key to Cro deprotonation in the ground state, so their removal promotes the neutral phenol form. The structure suggests T203 side chain rotates so that the hydroxyl group is further from Cro (4.8 Å *versus* 2.7 Å in sfGFP) and poorly orientated to H-bond to Cro (Fig. 2a). One possible explanation for the reorientated T203 side-chain is the presence of two local water molecules that can H-bond to the hydroxyl group of T203 (Fig. S3†); only one water is present in original sfGFP and positioned differently.

There is also a shift in the position of the loop housing residue 148, bringing it closer to the adjacent β-strand 6 resulting in the expulsion of a water molecule (Fig. 2b). As well as loss of the H-bond between the 148 imidazole and R168 backbone (as observed in other H148 mutants such as YFP H148G,^41^ deGFP1 (ref. 42) and GFPuv H148G^39^), the side chain of N146 changes positions so removing additional H-bonds to residues in strand 8 that links the two adjacent regions together (Fig. S4†). This appears to remove structural constraints on residues prior to azF148 so allowing the loop to close the distance to the adjacent strand 6. Such a local rearrangement has implications in terms of water molecule placement. Two ordered water molecules are observed for sfGFP, W^CRO^ and W^B^. W^CRO^ is a structurally conserved water molecule that plays a role in the proton transfer network,^37,43–45^ and similarly positioned in sfGFP^148azF^ (Fig. 2b). W^B^ is observed at a similar position in other GFP variants (*e.g.* EGFP,^43,46^ the S65T GFP variant^44^ and YFP^41^) but absent in sfGFP^148azF^, with the nearest water molecule ∼7.5 Å away. The two structured waters, along with the phenolic oxygen, may make a critical contribution to the H-bond/charge transfer network in original sfGFP (as has been proposed previously^42^) that promotes the phenolate ground state form which has been disrupted on mutation of H148 to azF.^34^ The potential role of this water channel and interaction network is discussed later.

### Fluorescence switching through azide–alkyne cycloaddition

Cu-free azide–alkyne cyclcoaddition is becoming a popular approach for biocompatible and bioorthogonal conjugation of biomolecules.^1,47,48^ However, little is known about the impact on protein structure and how any induced structural changes can be used to influence function, in a manner akin to natural PTM events. SPAAC is generally used to attach labels to proteins with no anticipated changes in structure and function but recent studies have indicated it can be used to control enzyme activity, presumably through active site blocking.^24,49^ Using sfGFP^148azF^ as a model system, we investigated if positive functional switching through propagated structural changes, a process routinely used in nature,^7^ can be achieved using a small easy to use “off-the-shelf” cyclooctyne. Residue 148 is ideally placed to act as modulation site due to its indirect role in chromophore fluorescence (*via* interaction networks). Additionally, AzF148 is only partially exposed (Fig. 1c; predicted SASA of 60.1 Å^2^; 33%) suggesting that the side-chain needs to be repositioned in order to accept an incoming adduct and form the triazole ring.

**Fig. 3:**
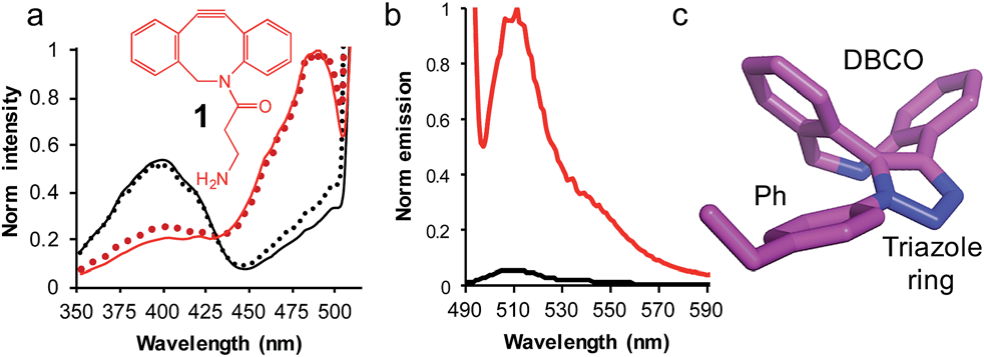
The use of SPAAC to switch sfGFP^148azF^ function (a) change in fluorescence excitation spectra *in vitro* (solid line) and *in vivo* (dotted line) in the absence (black) and presence (red) of DBCO-amine (**1**, inset). *In vivo* experiments represent fluorescence from intact cells after incubation of **1** with cell cultures for up to 8 h. (b) Emission spectra on excitation of 400 nm (black line) and 485 nm (red line) of isolated sfGFP^H148azF^ modified with **1**. The unmodified sfGFP^148azF^ emission spectra is shown in Fig. S1;† the major emission peak corresponds to excitation at 400 nm. (c) Determined structure of the DBCO moiety linked to azF148. The amino acid side chain (Ph), triazole link and DBCO are shown.

The simple strained alkyne molecule, dibenzocyclooctyne-amine (**1**, DBCO-amine, Fig. 3a) was chosen as it is one of the simplest commercially available DBCO compounds and can add new long range bonding capability (*e.g.* H-bonds *via* its primary amine and triazole groups) and is membrane permeable (*vide infra*).

Reacting sfGFP^148azF^ with **1** either *in vivo* or *in vitro* resulted in a shift in the major excitation (Fig. 3a) and absorbance (Fig. S1†) peaks from ∼395 nm to ∼490 nm. The major emission peaks on excitation at either wavelength was ∼511 nm, in line with emission from the phenolate anion form of the chromophore in the excited state (Fig. S1†). Modification *in vivo* is an important observation as it indicates that a small, uncharged DBCO adduct such as **1** is permeable through both cell membranes and cell wall of the Gram-negative *E. coli* to allow modification in the complex cell milieu. Modified sfGFP^148azF^ was successfully separated from unmodified protein; mass spectrometry confirmed the isolated species was protein with **1** attached (Fig. S5† and Table 2). The modification efficiency calculated using a fluorescent DBCO derivative (DBCO-585; Fig. S6†) was determined to be ∼75%. Interestingly, modification with DBCO-585 had little effect on the sfGFP chromophore absorbance spectrum (Fig. S6†); the 490 : 395 nm ratio in the absorbance spectrum suggests that the neutral phenol/anionic phenolate equilibrium population had not been significantly perturbed.

Emission of pure **1**-modified sfGFP^148azF^ was almost exclusively observed on excitation at ∼490 nm (Fig. 3b) despite the presence an absorbance peak at ∼400 nm (Fig. S1†). This represents a ∼95% loss of the emission at the less favorable 400 nm excitation, and a ∼3.5 fold increase in emission at the preferred 485 nm excitation. The quantum yield and overall brightness at the optimal 490 nm excitation significantly improved on modification (∼3 and ∼6 fold, respectively; Table 1). The quantum yield was also higher (85%) than the original sfGFP (75%) and other commonly used GFPs (*e.g.* EGFP 60%,^43^ EYFP 61%) on excitation at the preferred 490 nm.

### Structure of SPAAC modified sfGFP^148azF^

The crystal structure of SPAAC modified sfGFP^148azF^ (Table S1;† PDB code ; 5DY6) was determined, which is to our knowledge the first such structure of a SPAAC modified protein. The electron density around residue 148 benzyl-triazole-DBCO moiety was relatively well defined (Fig. S7†) but no clear electron density was observed for the amine protrusion suggesting that it is dynamic. The triazole ring lies ∼45° to the plane of the aromatic side chain with the octonyl ring puckered to form a boat conformation (Fig. 3c).

**Fig. 4:**
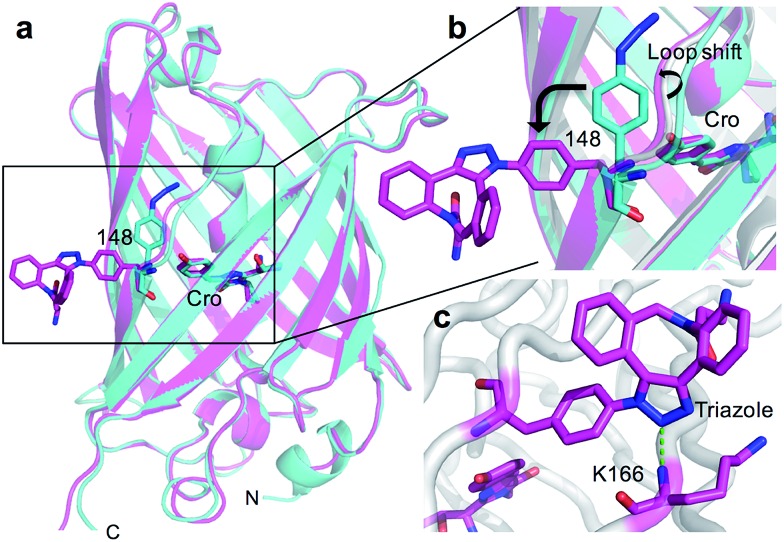
Structure of SPAAC modified sfGFP^148azF^. (a) Overlay of sfGFP^148azF^ pre (cyan) and post (magenta) modification with 1. (b) Close-up of the region around residue 148. Original sfGFP is coloured grey. (c) Putative H-bond (green dashed line) formed between the triazole link and K166.

The overall structure of sfGFP^148azF^+**1** is similar to the unmodified protein (Fig. 4a). The main structural changes are observed within the proximity of residue 148. Linkage of **1** to azF148 through the triazole results in the phenyl side chain rotating by ∼90° around the χ1 dihedral, with the aromatic group remaining in the same plane (Fig. 4b). The loop housing 148 also shifts to a position similar to that occupied by original sfGFP, regenerating the solvent channel and interaction network through to the chromophore (*vide infra*). A putative H-bond between the triazole central N atom and the backbone amide of K166 may play a role in stabilising the conformation of **1** and contribute to the loop shift (Fig. 4c). Thus, the general mechanism of action is local conformation changes that alter a critical non-covalent interaction network. This is akin to PTM mechanisms in nature such as phosphorylation.^7^ There are shifts in side chain placement in residues surrounding the chromophore, including T203 and E222 (Fig. S7†), two residue known to be important in defining chromophore protonation state.^36^ Subtle perturbations in the chromophore environment can influence the relative populations of the phenolate and neutral phenol forms.

### Molecular basis of photochemical switching

**Fig. 5:**
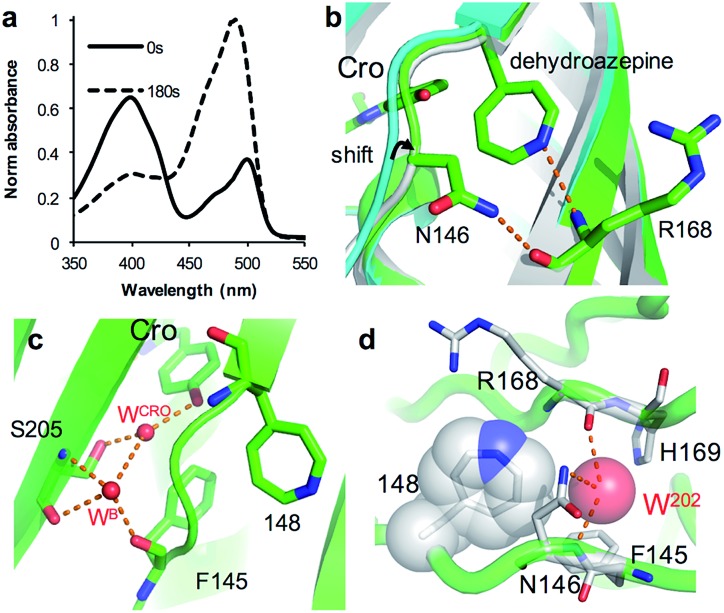
Structural changes on irradiation of sfGFP^148azF^. (a) Absorbance spectrum before (full line) and after 180 s irradiation (dashed line). (b) Structural overlay of original sfGFP (grey), dark state sfGFP^148azF^ (cyan) and light state sfGFP^148azF^ (green). The proposed dehydroazepine and potential H-bonds are shown. (c) The proposed water channel in light state sfGFP^148azF^. Water molecules (W) are shown as red spheres. The corresponding water molecules in original sfGFP and dark state sfGFP^148azF^ are shown in Fig. 2b. (d) Placement of water molecule (O^202^, red sphere) in relation to dehydroazepine at residue 148 (grey).

As an alternative to SPAAC modification, phenyl azide photochemistry can be used to modulate GFP fluorescence.^16,18^ Classically, phenyl azide photochemistry has been used in biology as a crosslinking reagent.^11,12^ The photochemistry of phenyl azide is relatively complex with several routes available on loss of N_2_ and formation of singlet nitrene including bond insertion, reduction to the phenyl amine and ring expansion to a dehydroazepine (Scheme S1†). Different positions within the same protein can elicit distinct photochemical events and thus functional effects.^16–18^ As reported briefly before by our group,^18^ irradiation of sfGFP^148azF^ switches the major absorbance and excitation peak from ∼395 nm to ∼490 nm (Fig. 5a and Table 1). The structure of the starting ‘dark’ form of sfGFP^148azF^ provides an explanation for this switch in electronic excitation due to disruption of the charge-transfer system that promotes the phenolate form (Fig. 2a; *vide supra*). To understand the molecular basis of how azF photolysis switches sfGFP fluorescence, the structure of the *in crystallo* irradiated sfGFP^148azF^ (termed light state) was determined (Table S1;† PDB code ; 5BTT). Two molecules comprise the unit cell with overall structures of the two being very similar (RMSD of 0.33 Å). One apparent difference between the two is the electron density observed for residue 148 (Fig. S8†). The electron density in chain A strongly indicates an extended ring structure with no clear evidence of protrusion, interpreted as the formation of a dehydroazepine (Scheme S1† and ref. 50). In the second molecule (chain B), the electron density best fits a six membered ring with an extension at the *para* position, interpreted as a phenyl group with at least a single nitrogen projecting from the *para* position suggesting the formation a phenyl amine or possibly an azirine intermediate stabilised within the static crystal; we cannot rule out the formation of an azepinone, where the protrusion may represent a ketone (Fig. S8; *vide infra*; Scheme S1†). We have previously observed that replacing residue 148 with the ncAA aminophenylalanine (introducing the equivalent of a phenyl amine group)^18^ does not result in the spectral shift observed for sfGFP^148azF^. Thus, combined with mass spectrometry data (Table 2 and Fig. S9†) the phenyl amine is unlikely to be the final end-product. From here on in, we will focus on the proposed dehydroazepine ring form.

Photoreaction of azF148 induces conformational changes in the protein; shifts in backbone and side chain placement coupled with structured water networks are likely to drive excitation switching on photolysis. As with modification with **1**, a small (∼0.6 Å) but significant shift in the backbone position of residues N-terminal to residue 148, to a position similar to that occupied in sfGFP was observed on conversion to the light state (Fig. 5b). The nitrogen in the dehydroazepine ring together with the N146 side chain potentially form H-bonds with the R168 backbone amide and carbonyl groups respectively, so contributing to the backbone shift. This generates a water channel and network present in original sfGFP directly to the chromophore, with the two ordered water molecules W^CRO^ and W^B^ (see Fig. 2b) also observed in the light form of sfGFP^148azF^ (Fig. 5c). The backbone shift is critical in allowing W^B^ access to the β-barrel in light state sfGFP^148azF^; the two waters, along with the phenolic oxygen, make up a potential local H-bond network. As well as interacting with water W^Cro^, W^B^ can potentially act as a H-bond acceptor for the S205 backbone NH and H-bond donor to the F145 backbone carbonyl oxygen (Fig. 5c). The crystal structure indicates that the T203 side chain retains the same geometry in both the dark and light state sfGFP^148azF^, placing it in a different orientation to sfGFP. This change in side chain geometry does affect the H-bond contacts as noted above. Otherwise, the residues within 4 Å of the chromophore take up very similar positions in original sfGFP and light state sfGFP^148azF^ (Fig. S10†).

When using phenyl azide chemistry in the context of proteins as a non-discriminate photocrosslinking reagent, the presumption is that nucleophilic attack by the dehydroazepine intermediate on primary amines (*e.g.* lysines) will predominate. In the case of azF148, there is no suitable amino acid partner for the nitrene to react with through the pathways outlined in Scheme S1,† thus the likely route is *via* dehydroazepine.^51^ As the dehydroazepine is normally considered to be a reaction intermediate, especially if local nucleophiles are present,^51^ we determined the mass change on photolysis in solution conditions. The measured mass was 27 865 Da (Fig. S9†), a decrease of ∼12 Da compared to the dark form (Table 2). The first stage of photolysis is loss of N_2_ (mass decrease of ∼28 Da), which can then undergo various reactions with small subsequent changes in mass (Scheme S1;† Table 2). The observed mass suggests that the dehydroazepine reacts further to gain +16 Da, with the most likely outcome being formation of an azepinone.^51^ We postulate that a localized structured water molecule (W^202^) could act as the nucleophile (Fig. 5d). While water is considered a weak nucleophile, the highlighted water molecule can potentially form H-bonds with neighbouring amino acid residues which can increase its nucleophilicity.

### Different chemical routes; similar molecular mechanism

**Fig. 6:**
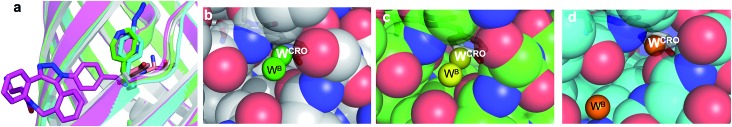
Comparison of conformational changes on modification of residue 148. (a) Structural overlay of original sfGFP (grey), dark-state sfGFP^148azF^ (cyan), sfGFP^148azF^+**1** (magenta) and irradiated sfGFP^148azF^ (green). Changes in the solvent channel and observed water molecules through to the chromophore for (b) original, wt sfGFP, (c) and irradiated, light state sfGFP^148azF^, and (d) dark-state sfGFP^148azF^. The protein structure is shown as van der Waals atomic radii (sphere) representation, the chromophore is shown as stick representation in (b–d). The water molecules are shown as solid spheres, labelled as in Fig. 2 and 5. The water molecules are scaled to 0.5 that of the standard oxygen van der Waals radius to aid with clarity. The equivalent sfGFP^148azF^+**1** structure is shown in Fig. S11.†

The molecular basis of functional switching in sfGFP^148azF^ was similar for both the photochemical and SPAAC reactions: local changes to the interaction network that contribute towards protein function. The loop housing residue 148 shifts on substitution of His for azF (Fig. 6a), with one of the main consequences being closure of solvent channel and breaking the water interactions network through to the chromophore; it is narrower in sfGFP^148azF^ with only a single water observed in the channel (Fig. 2). On modification of azF148, the backbone position reverts to a similar position occupied by original sfGFP and the water molecule network reforms (Fig. 6). Critical to loop repositioning is the potential contribution of the new H-bonding groups generated on either addition of **1** (Fig. 4) or formation of the expanded ring (Fig. 5); the new chemical groups are thus contributing to the reorganisation of the non-covalent network. Loop repositioning is likely aided by reorientation of the N146 side chain to a similar position occupied in original sfGFP (Fig. S4†) so promoting H-bonding with the adjacent strand 8. These new interactions make up for the loss of the original H-bonds lost on mutation of H148 to azF. Visual inspection and analysis using CAVER^52^ shows that the channel and water interaction network is similar in terms of pathway, width and residue positioning in original sfGFP, sfGFP^148azF^+**1** and irradiated sfGFP^148azF^ (Fig. 6b, c and S11†). The equivalent features are absent in dark state sfGFP^148azF^ (Fig. 6d and S11†). The region around residues 146 and 148 is considered a key proton “exit point” within the charge transfer network;^37,45^ disruption of the proton exit point (as with dark state sfGFP^148azF^) should promote the neutral form of the chromophore and its reestablishment should promote the anionic chromphore. Two waters (Fig. 2, 5 and 6), along with the phenolic oxygen, could make up a potential local H-bond and proton shuttling network that contribute to the chromophore protonation state and thus the excitation properties. We (*vide supra*) and others^37,42,45^ have already discussed the importance of these structured water molecules in defining the proton transfer network. Thus, it is possible to use non-natural reaction handles, such as azF, to modulate a protein interaction network through different chemical routes. Given the current importance of optogenetics^53,54^ and non-native PTM,^1,6^ additional and potentially versatile approaches to regulating protein function that are understood at the molecule level would be welcome. By targeting induced structural changes (as opposed to simple active site blocking), allosteric and non-catalytic bioprocesses may become accessible to nnPTM events using ncAAs, so expanding its use.

## Conclusion

We have demonstrated the versatility of genetically encoded phenyl azide chemistry to positively switch protein function through two different non-native PTM events: photolysis and bioorthogonal modification. Using sfGFP as a model to aid our molecular understanding, the observed mechanism of action for both approaches is indirect through altering the local interaction network that propagate to the functional centre [the chromophore]. In both cases, the nnPTM changes local H-bonds networks but in distinct ways. As local interaction networks are central to protein function there is scope for genetically encoded phenyl azide chemistry to be applied more generally to modulate protein activity beyond classical photocrosslinking and simple labelling. The structural knowledge, outlined here, of the mechanism of action will help understand the molecular pathways open. This information can in turn be funnelled into *in silico* design^24^ so aiding future protein engineering efforts. Additional molecular details in other protein systems will undoubtedly improve such engineering endeavours and provide insights into how the protein molecular environment dictates an individual pathway, especially with regards to photochemical endpoints. This is an area we are currently exploring.

## Methods

### Protein engineering and production

The TAG replacement mutagenesis and the sfGFP^148azF^ protein production was performed essentially as described previously^18^ and reported in detail in the ESI.†

### Photolysis and click chemistry

The sfGFP^148azF^ variant in 50 mM PBS was irradiated with UV light using a UVM-57 handheld UV lamp (UVP). Proteins were irradiated for up to 30 minutes at a distance of 1 cm from the sample, sampling a range of UV wavelengths (275–380 nm) at 4 watts, as defined by the light source. SPAAC reactions were performed on pure protein using a five-fold molar excess of DBCO-amine or DBCO-585 (click chemistry tools) to protein in PBS. Reactions were left for up to 8 h at room temperature. Excess modification was removed using a Vivaspin 100 Sample Concentrator Unit 10 000 MWCO (GE Life Sciences). Click-modified protein was purified from unmodified protein by anion exchange chromatography using MonoQ 5/50 GL column (GE Life Sciences) at a flow rate of 0.5 mL min^–1^. The proteins were eluted using a concentration gradient of NaCl, from 0 mM to 500 mM over 20 column volumes. Elution fractions containing modified and unmodified protein were analysed by SDS-PAGE and fluorescence spectroscopy. Click reactions with whole cell samples were performed by the mixing of DBCO-amine and cells, followed by incubation at room temperature for up to 8 h. Cells were then pelleted by centrifugation and washed with PBS. Cells were standardized to an OD of 0.1 in TNG buffer (50 mM Tris, 150 mM NaCl, 10% (v/v) glycerol, pH 8) for fluorescence measurements.

### Spectroscopic analysis

Fluorescence and absorbance spectroscopy was performed as outlined in the ESI.† Mass spectrometry was performed using by LC/MS-TOF using a Waters Synapt G2-Si QT as outlined in the ESI.†

### X-ray crystallography

Purified protein was buffer exchanged into 50 mM Tris, 150 mM NaCl, pH 8, and concentrated to 10 mg mL^–1^. Crystal formation was screened using the sitting drop vapour diffusion method across a variety of buffer conditions. Screens were set up in duplicate, at 4 °C and at 25 °C. Drops were set up with equal volumes of protein and precipitant solution (0.2 μL). The condition that produced crystals are outlined in ESI.† Two crystals of sfGFP^148azF^ were observed. One was used for the dark state structure and the second collected and irradiated to generate the light state form. Data were collected at the Diamond Light Source (Harwell, UK). The structure was determined as outlined in the ESI.†
