# Supplementary material for: Molecular basis for functional switching of GFP by two disparate non-native post-translational modifications of a phenyl azide reaction handle
Source: Chem Sci. 2016 Jun 29;7(10):6484–91. doi: 10.1039/c6sc00944a (PMC5355941; doi:10.1039/c6sc00944a)
Supplement: Supplementary file 1 [file SC-007-C6SC00944A-s001.pdf]

Electronic Supplementary Material (ESI) for Chemical Science.
This journal is © The Royal Society of Chemistry 2016

Positive functional switching of GFP by two disparate non-native post-translational modifications of a single reaction handle.

Andrew M. Hartley[a], Harley L. Worthy[a], Samuel C. Reddington, Pierre J. Rizkallah, D. Dafydd Jones.

Supporting information

Site-directed mutagenesis

The amber stop codon mutation at position 148 in the sfGFP gene (in pBAD/HisA) was introduced as described in Reddington et al, 2013 [1]. Briefly, the Phusion site-directed mutagenesis PCR protocol (NEB) was used with a forward primer (5'-TAGAATGTGTATATTACCGCCGATAAACAGAAACAGAAAAATGG-3') and the reverse primer (5'-GCTGTTGAAATTATATTCCAGTTTATGACCCAGAATG-3').

Protein production

Protein production was performed in *E. coli* TOP10 cells (Invitrogen) using ZYM5052 auto-induction medium. ZYM-5052 medium (Studier, 2005) was composed of 1 % (w/v) Tryptone, 0.5 % (w/v) Yeast Extract, 0.5 % (v/v) glycerol, 0.05 % (w/v) glucose, 0.2 % (w/v) lactose, 25 mM Na_2HPO_4, 25 mM KH_2PO_4, 50 mM NH_4Cl, 5 mM Na_2SO_4, 2 mM $MgSO_4$, 1 × trace metals and 0.05 % (w/v) L-arabinose in ultra-pure water. 1 × trace metal mixture contained 4 µM $CaCl_2$, 2 µM $MnCl_2$, 2 µM $ZnSO_4$, 0.4 µM $CoCl_2$, 0.4 µM $CuCl_2$, 0.4 µM $NiCl_2$, 0.4 µM Na_2MoO_4, 0.4 µM H_3BO_3, and 10 µM $FeCl_3$ in ultra-pure water. TOP10 cells were transformed with both pBAD-sfGFPHis^{148azF} and pDULEazidoRS, which contains an engineered *Methanocaldococcus jannascii* tyrosyl-tRNA-synthetase and tRNA^{CUA} orthogonal pair that can recognise azF. *p*-Azido-L-phenylalanine (azF) (Bachem) was prepared prior to use by dissolving the appropriate weight of powdered amino acid in ultra-pure water and titrating in 1 M NaOH. AzF was added to growth media to a final concentration of 1 mM. Single colonies were used to inoculate 10 mL LB containing 100 µg/mL ampicillin and 25 µg/mL tetracycline, and the culture grown to saturation. Expression cultures of ZYM5052 (+ Amp, + Tet) were inoculated with the saturated starter culture diluted 1/200 and grown at 37°C for 20 hours. Protein production and solubility was confirmed using SDS-PAGE analysis. Cells were harvested by centrifugation and lysed in sodium phosphate buffer (100 mM, 300 mM NaCl; pH 8) by French Press. Protein samples were handled under predominantly red-spectrum light to prevent azF from photoreaction.

Protein purification

Proteins were purified by Ni-affinity chromatography using HisTrap™ HP column (GE Life Sciences) attached to an ÄKTApurifier (GE Life Sciences). Samples were bound to the column in PBS buffer (50 mM sodium phosphate, 100 mM NaCl, pH 8.0) at a flow rate of 1 mL/min. Protein elution was initiated by gradual increase in concentration of imidazole (0-250 mM) over 20 column volumes at a flow rate of 3 mL/min. Elution of contaminants was monitored by absorption at 280 nm, while elution of sfGFP (and azF variants) by absorption at 485 nm. Elution fractions containing protein were pooled and desalted into PBS buffer using Vivaspin 20 Sample Concentrator Unit 10,000 MWCO (GE Life Sciences).

Click-modified proteins were purified from unmodified proteins by anion exchange chromatography using MonoQ 5/50 GL column (GE Life Sciences) in Tris buffer (50 mM) at flow rate of 0.5 mL/min. The proteins were eluted using a concentration gradient of NaCl, from 0 mM to 500 mM over 20 column volumes. Elution fractions containing modified and unmodified protein were analysed by SDS-PAGE and fluorescence spectroscopy.

Photolysis and Click Chemistry

$sfGFP^{His148azF}$ in 50 mM PBS was irradiated with UV light using a UVM-57 handheld UV lamp (UVP). Proteins were irradiated for up to 30 minutes at a distance of 1 cm from the sample, sampling a range of UV wavelengths (275 – 380 nm) at 4 watts, as defined by the light source.

Strain promoted azide-alkyne cyclo-addition (SPAAC) reactions were performed on pure protein using a five-fold molar excess of DBCO-amine or DBCO-585 (Click Chemistry Tools) to protein in PBS. Reactions were left overnight at room temperature. Excess modification was removed using a Vivaspin 100 Sample Concentrator Unit 10,000 MWCO (GE Life Sciences).

In vivo click reactions were performed by adding DBCO-amine (20 μM) to an *E. coli* culture after $sfGFP^{148azF}$ induction. The cultures were left at 25°C for up to 8 hr. The cells were then pelleted by centrifugation and washed with PBS. Cells were standardized to an OD of 0.1 in TNG buffer (50 mM Tris, 150 mM NaCl, 10% (v/v) glycerol, pH 8) for fluorescence measurements outlined below. A control experiment without addition of DBCO-amine to cell cultures was also performed.

Fluorescence and Absorbance Spectroscopy

Fluorescence spectroscopy was performed using a Varian Cary Eclipse spectrophotometer. Spectra were recorded at a scan rate of 600 nm/min with a slit width of 5 nm. Emission spectra were recorded up to 650 nm from a single excitation wavelength (485 nm). Excitation spectra were recorded by measuring emission at 511 nm over a range of excitation wavelengths from 350 to 511 nm. For whole cell fluorescence, cells were pelleted and resuspended in TNG buffer (50 mM Tris-HCl, 150 mM NaCl, 10% (v/v) glycerol, pH 8) to an OD of 0.1. Pure proteins were analysed at a concentration of 1 μM in a 5 × 5 mm QS quartz cuvette (Hellma, Müllheim, Germany).

Quantum yields of $sfGFP^{His148azF}$ were determined by comparison with fluorescein, a reference with a known quantum yield at a similar wavelength (496 nm). $sfGFP^{His148azF}$ and fluorescein were diluted to an A_{485} of 0.05 and emission spectra were recorded at the excitation maxima of $sfGFP^{His148azF}$ and integrated between 5 nm above the excitation wavelength and 650 nm, and then inserted into the equation.

$$\Phi_x = \Phi_R \cdot \left(\frac{\mathrm{Int}_x}{\mathrm{Int}_R}\right) \cdot \left(\frac{\eta_x^2}{\eta_R^2}\right)$$

Φ is the quantum yield, *Int* is the integrated emission spectrum and η is the refractive index of the solvent. The subscripts x and R refer to the sample and reference respectively. Brightness was determined as a product of molar extinction coefficient and quantum yield.

For absorbance spectroscopy, purified protein samples were diluted in PBS buffer at 20μM concentration and placed in a 10 × 10 mm QS quartz cuvette. UV-vis absorbance spectra were recorded at 20°C using HP 8452A Diode Array Spectrophotometer (Hewlett Packard).

Mass Spectrometry

Proteins were buffer exchanged into water and concentrated using Vivaspin 100 Sample Concentrator Unit 10,000 MWCO (GE Life Sciences) to 10 μM. Mass spectra were recorded by LC/MS-TOF using a Waters Synapt G2-Si QT acquiring

data from 200-2000 Da in positive Electrospray ionisation mode using Leucine Enkephalin as the Lock mass. Proteins were passed through a Waters Acquity UPLC CSH 130 C18 held at 80°C and eluted using a gradient of acetonitrile, from 95% water with 0.1% formic acid to 95% acetonitrile with 0.1% formic acid over 5 minutes with an average injection volume of 2 μl. The data was processed using MassLynx 4.1 and deconvoluted using Maximum Entropy 1 add-on software.

X-ray crystallography

Purified $sfGFP^{148azF}$ was buffer exchanged into 50 mM Tris, 150 mM NaCl, pH 8, and concentrated to 10 mg/ml. Crystal formation was screened using the sitting drop vapour diffusion method across a variety of pH conditions (4-9 in steps of 0.5) and a variety of $(NH_4)_2SO_4$ concentrations (50-90% saturation in steps of 10%). Screens were set up in duplicate, at 4°C and at 25°C. Drops were set up with equal volumes of protein and precipitant solution (0.2 μl). The condition that produced crystals was 50 mM MMT (Malic acid, MES, Tris), 60% saturation with $(NH_4)_2SO_4$ (2.52 M), pH 8.5, which yielded two crystals. 1 mM ethylene glycol was added as a cryo-protectant before harvesting one of the crystals. The second crystal was then irradiated for 60 minutes using a UVM-57 handheld UV lamp (UVP). This second crystal was then harvested in the same manner. The condition that produced crystals of DBCO-amine modified $sfGFP^{148azF}$ was 0.1M PCTP Buffer, pH 6.0, 25% PEG 1500. All crystal screens were set up using an Art Robbins Phoenix robot, supplied by Alpha Biotech, U.K. The volume of the precipitant in the deep wells was 60 μl, and the drop contained 0.2 μl protein solution with 0.2 μl well solution. The tray was kept at 294°K.

Data were collected at the Diamond Light Source (Harwell, UK). Data were reduced using the XIA2 package [2] assigned a space group using POINTLESS [3], scaled using SCALA [3] and merged using TRUNCATE [4]. Structures were solved by molecular replacement with PHASER, using a previously determined sfGFP structure (PDB code 2B3P). Structures were then adjusted manually using COOT [5] and refined by TLS restrained refinement using RefMac [6]. All the above programs were accessed via the CCP4 package (http://www.ccp4.ac.uk/) [4].

Table S1. Data reduction and refinement statistics

	Dark	Light	DBCO
**Data collection/reduction statistics**			
Wavelength (Å)	0.92	0.92	0.97623
Beamline	Diamond I04-1	Diamond I04-1	Diamond I03
Space group	$P4_32_12$	$P4_32_12$	$P2_12_12_1$
a (Å)	135.6	135.14	42.98
b (Å)	135.6	135.14	89.38
c (Å)	69.23	69.56	122.37
Resolution range (Å)	42.88-2.03	56.24-2.14	72.18-2.66
Total reflections measured	600,948(42,383)	525,718(37,806)	99,142
Unique reflections	42,237 (3082)	36,124 (2,621)	14,111
Completeness (%) (last shell)	100 (100)	100 (100)	99.2 (99.4)
I/σ (last shell)	16.7 (3.9)	22.9 (4.0)	15.0 (1.1)
R(merge)[a] (%) (last shell)	10.3 (72.6)	7.9 (68.8)	5.8 (199.6)
B(iso) from Wilson ($Å^2$)	39.4	42.2	100.4
**Refinement statistics**			
Non-H atoms	4,079	4,002	3,658
Solvent molecules	350	274	2
R-factor[b] (%)	17.2	17.1	19.4
R-free[c] (%)	20.4	20.7	29.0
Rmsd bond lengths (Å)	0.018	0.018	0.016
Rmsd bond angles (°)	2.029	2.059	2.113
**Ramachandran Plot Statistics**			
Core region (%)	97.3	97.03	94
Allowed region (%)	2.47	2.97	5
Additionally allowed region (%)	0	0	0
Disallowed Region (%)	0.22	0	1

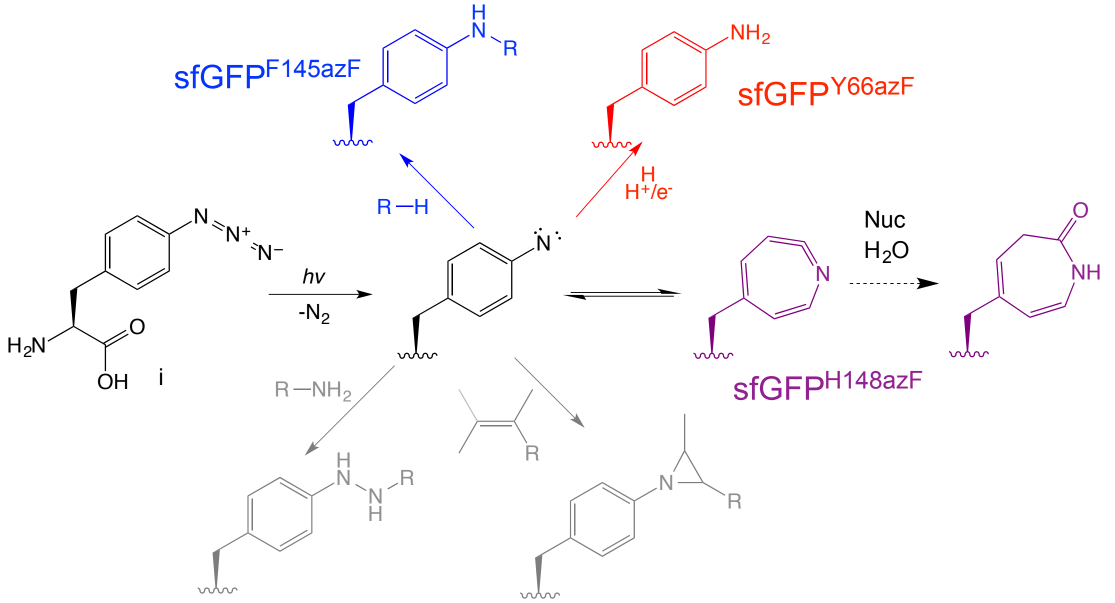

Scheme S1. Potential routes of the phenyl azide photoconversion after irradiation with UV light. Two routes previously suggested [1] to impact the fluorescence and photoactivity of sfGFP are highlighted (sfGFP^{Y66azF} and sfGFPF^{145azF}), as well as the proposed mechanism at H148.

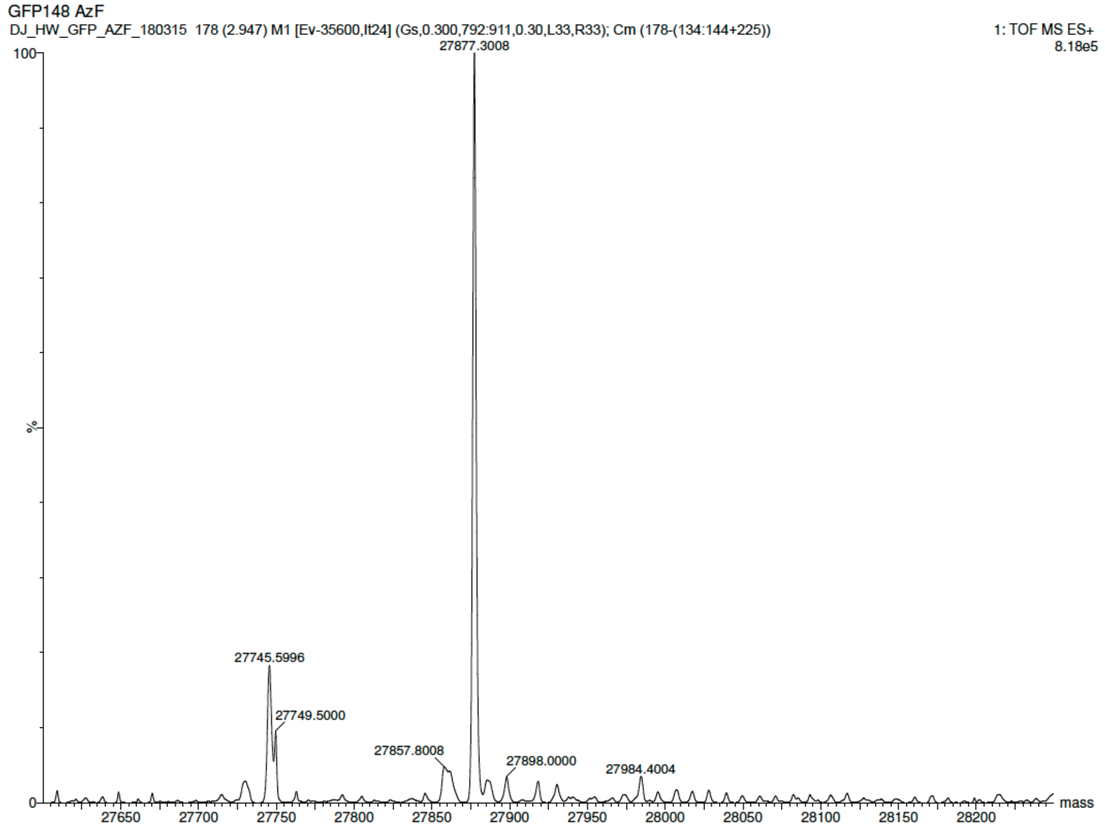

Figure S1. Mass spectrum of sfGFP^{148azF}. The measured and calculated masses are 27877 and 27879 Da, respectively.

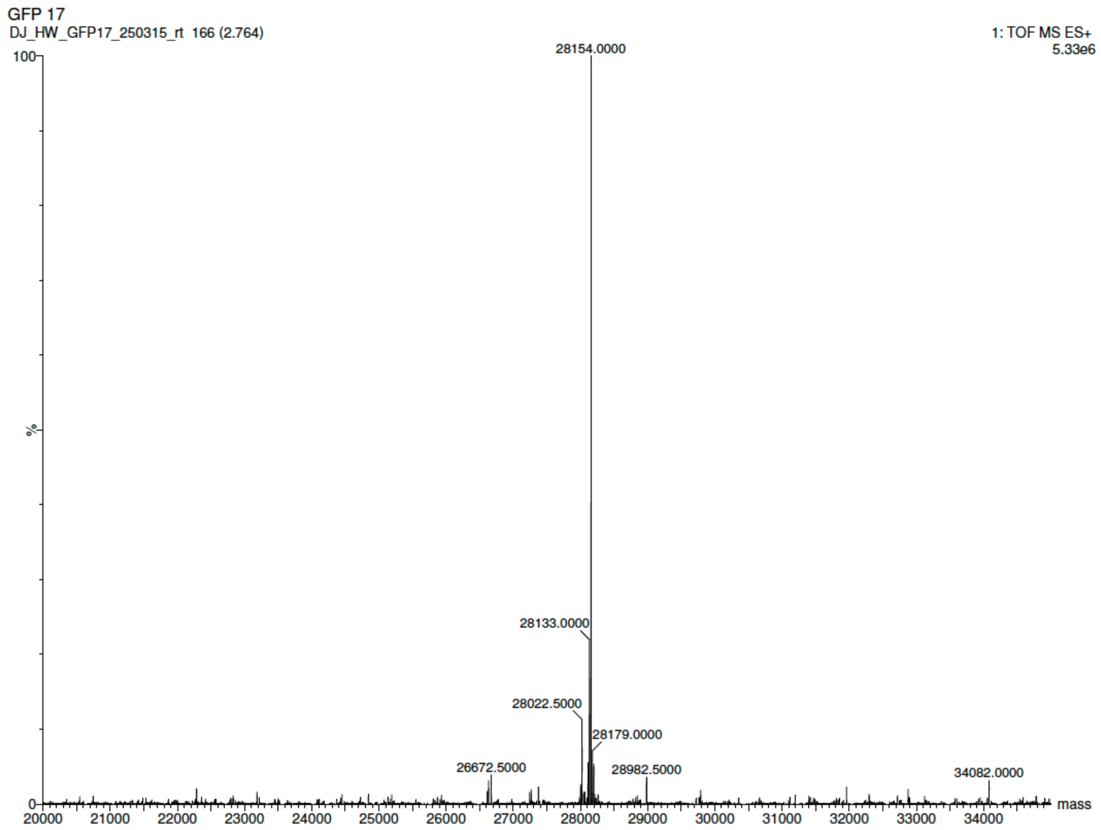

Figure S2. Mass spectrum of sfGFP^{148azF} modified with DBCO-amine. The measured and calculated masses are 28154 and 28155 Da, respectively.

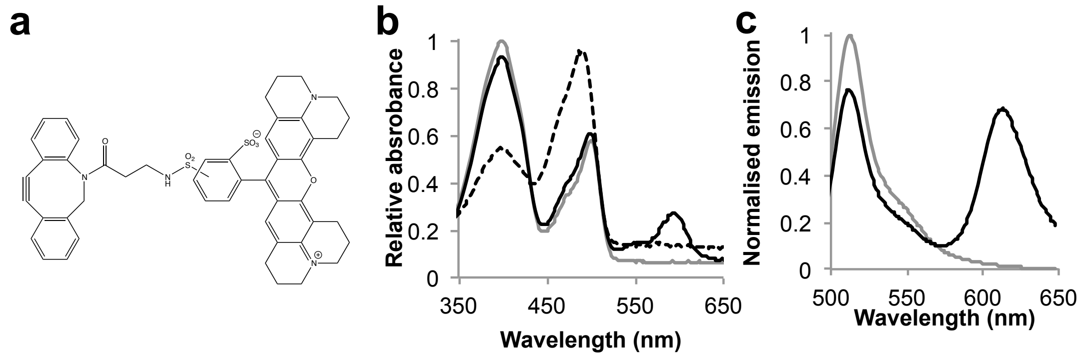

Figure S3. Modification of sfGFP^{azF148} with DBCO-585. (a). Chemical structure of DBCO-585 based on the Texas Red fluorescent probe. (b). Absorbance spectrum of unmodified sfGFP^{H148azF} (grey), sfGFP^{H148azF}-585 (black) and sfGFP^{H148azF}-amine (dashed). (c). Fluorescence emission of unmodified sfGFP^{H148azF} (grey) and sfGFP^{H148azF}-585 (black).

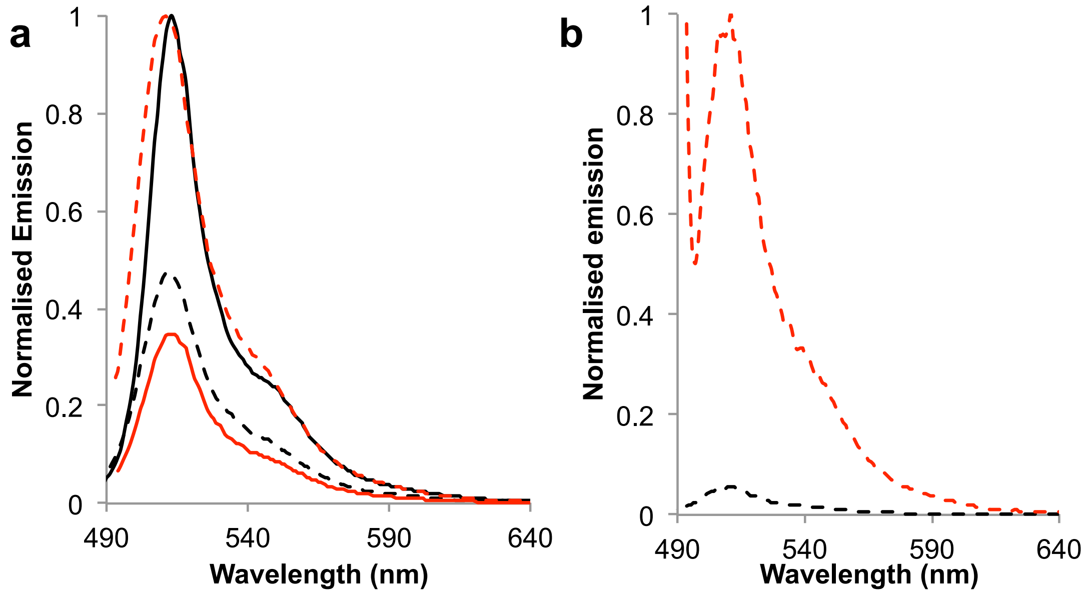

Figure S4. Effect on fluorescence emission on modification with DBCO-amine. (a). Fluorescence emission of unmodified sfGFP^{H148azF} (solid lines) and the sfGFP^{H148azF}-DBCO-amine reaction mixture (dashed lines). Emission measured upon excitation at 400 nm (black lines) and 485 nm (red lines). (b). Emission upon excitation at 400 nm (black line) and 485 nm (red line) of purified sfGFP^{H148azF} modified with DBCO-amine. Mass spectroscopy (Figure S2) confirmed that no unmodified protein was present.

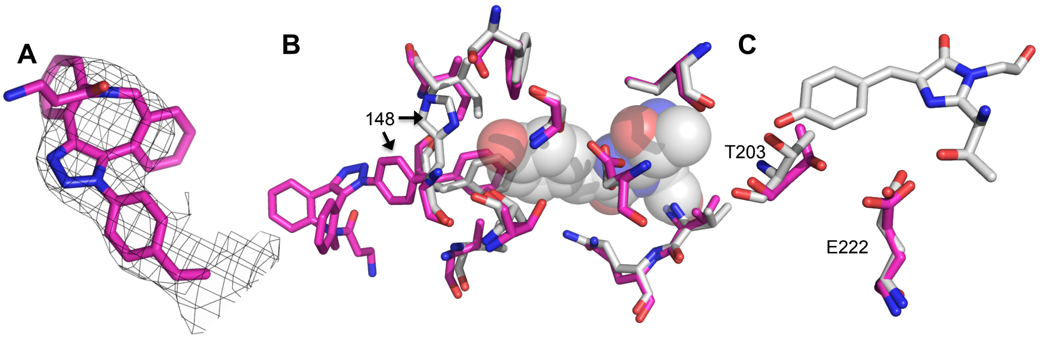

Figure S5. (A) Electron density around the DBCO-148 linkage. Structural comparison of (B) residues surrounding the chromophore and (C) key residues (T203 and E222) that contribute towards promoting the phenolate form. Native sfGFP is coloured grey and DBCO-amine modified sfGFP^{148azF} is coloured magenta.

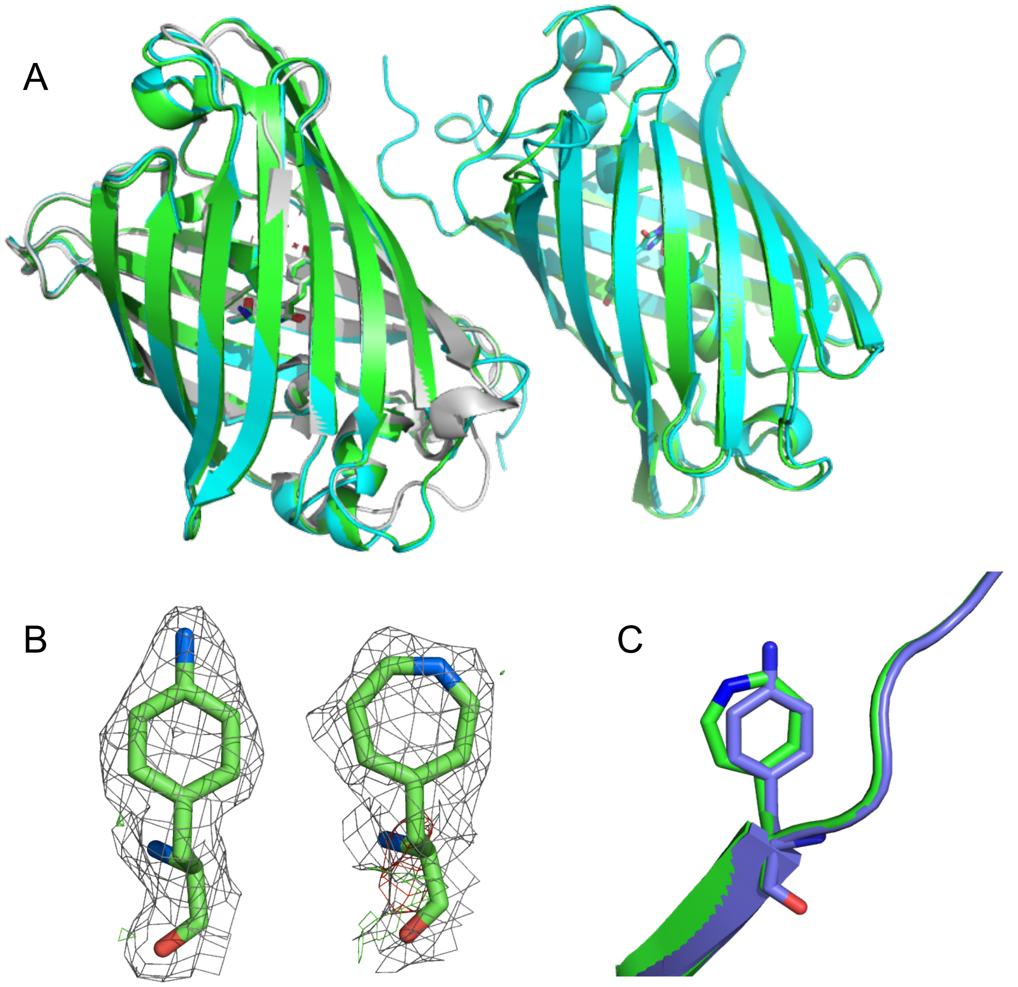

Figure S6. Effect of UV irradiation on the structure of sfGFP^{148azF}. (A). Comparison of native sfGFP (grey), sfGFP^{H148azF} dark state (green) and sfGFP^{H148azF} light state (cyan). (B) Comparison of the electron density surrounding residue 148 in the two molecules of the asymmetric unit of the light (irradiated) state of sfGFP^{H148azF}. One molecule was modelled with a single nitrogen projecting from the *para* position of the phenyl ring, whereas the second was modelled with a 7-membered dehydrozepine ring. (C) Overlay residue 148 of two molecule (A and B chain).

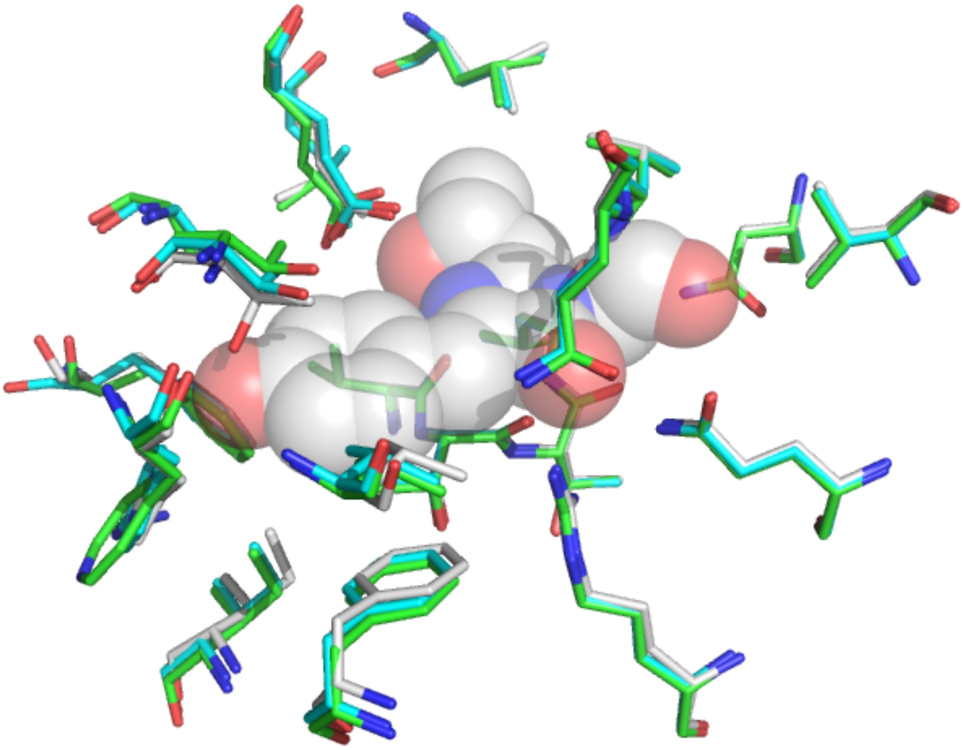

Figure S7. Alignment of all residues within 4 Å of the chromophore in sfGFP (grey), sfGFP^{148azF} dark state (cyan) and sfGFP^{148azF} light state (green). The chromophore is shown as grey spheres. In the crystal structure of dark state sfGFP^{148azF} (PDB code 5BT0), there is electron density only until the first nitrogen; the full azide group is not visible. This is a common phenomenon of azide group due to its mobility, resulting in multiple conformations existing. Mass spectrometry was used to confirm the presence of the full azide (Fig S1).

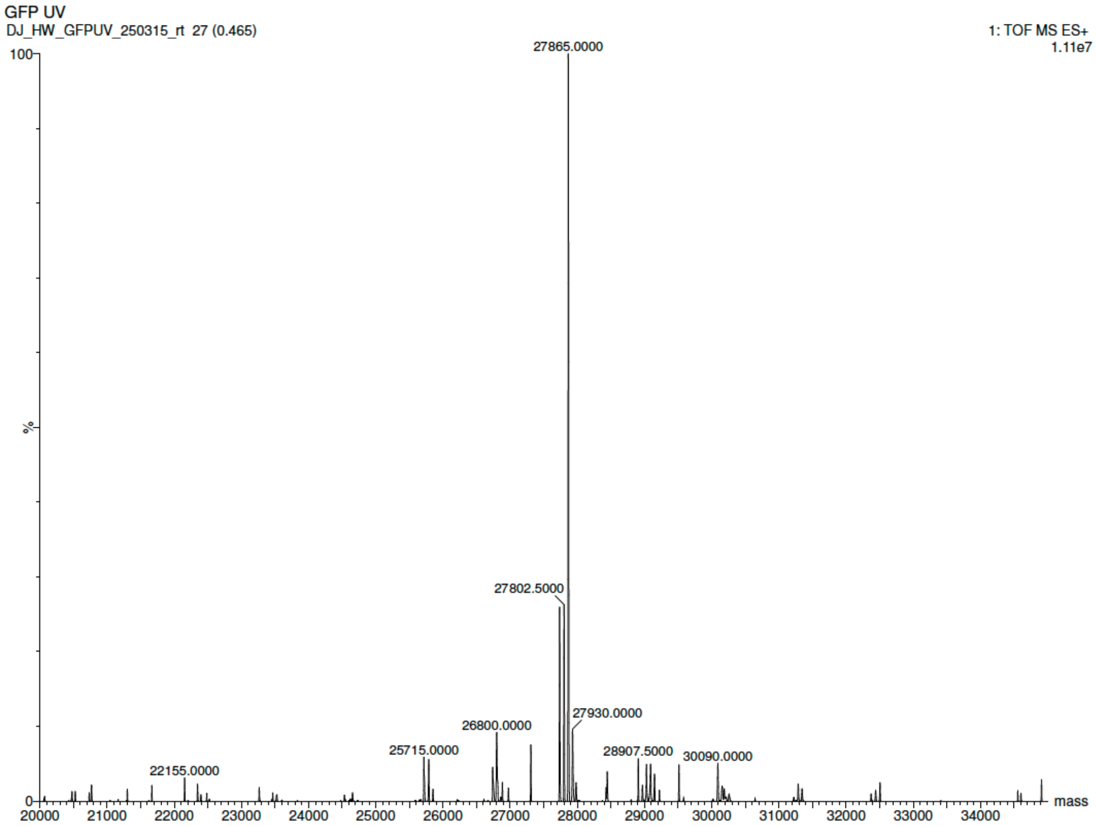

Figure S8. Mass spectrum of UV-irradiated sfGFP^{azf148}. A list of the masses of potential products of photoconversion is given in Table 2.

Supporting References

1. Reddington, S. C.; Rizkallah, P. J.; Watson, P. D.; Pearson, R.; Tippmann, E. M.; Jones, D. D., Different photochemical events of a genetically encoded phenyl azide define and modulate GFP fluorescence. *Angewandte Chemie* **2013,** *52* (23), 5974-7.
2. Winter, G., xia2: an expert system for macromolecular crystallography data reduction. *J Appl Crystallogr* **2009,** *43*, 196-190.
3. Evans, P., Scaling and assessment of data quality. *Acta Crystallogr D Biol Crystallogr* **2006,** *62* (Pt 1), 72-82.
4. Bailey, S., The Ccp4 Suite - Programs for Protein Crystallography. *Acta Crystallogr D Biol Crystallogr* **1994,** *50*, 760-763.
5. Emsley, P.; Cowtan, K., Coot: model-building tools for molecular graphics. *Acta Crystallogr D Biol Crystallogr* **2004,** *60* (Pt 12 Pt 1), 2126-32.
6. Murshudov, G. N.; Vagin, A. A.; Dodson, E. J., Refinement of macromolecular structures by the maximum-likelihood method. *Acta Crystallogr D Biol Crystallogr* **1997,** *53* (Pt 3), 240-55.
